# Oropharyngeal colostrum immunotherapy and nutrition in preterm newborns: meta-analysis

**DOI:** 10.11606/s1518-8787.2021055003051

**Published:** 2021-12-03

**Authors:** Michelle de Santana Xavier Ramos, Camilla da Cruz Martins, Elivan Silva Souza, Graciete Oliveira Vieira, Isaac Suzart Gomes-Filho, Ana Claudia Morais Godoy Figueiredo, Maurício Gomes Pereira, Simone Seixas da Cruz

**Affiliations:** I Universidade Estadual de Feira de Santana Departamento de Saúde Feira de Santana BA Brasil Universidade Estadual de Feira de Santana. Departamento de Saúde. Feira de Santana, BA, Brasil; II Universidade Federal do Recôncavo da Bahia Centro de Ciências da Saúde Santo Antônio de Jesus BA Brasil Universidade Federal do Recôncavo da Bahia. Centro de Ciências da Saúde. Santo Antônio de Jesus, BA, Brasil; III Universidade de Brasília Faculdade de Ciências da Saúde Brasília DF Brasil Universidade de Brasília. Faculdade de Ciências da Saúde. Brasília, DF, Brasil; IV Secretaria de Estado de Saúde do Distrito Federal Brasilia DF Brasil Secretaria de Estado de Saúde do Distrito Federal. Brasilia, DF, Brasil; V Universidade de Brasília Escola de Medicina Brasilia DF Brasil Universidade de Brasília. Escola de Medicina. Brasilia, DF, Brasil

**Keywords:** Infant, Very Low Birth Weight, Infant, Premature, Immunotherapy, Colostrum, Systematic Review

## Abstract

**OBJECTIVE:**

To investigated the effect of oropharyngeal colostrum immunotherapy in reducing the time required for very low birth weight preterm newborns (VLBW-PTNB: < 1,500g and < 37 weeks) to achieve full enteral nutrition.

**METHODS:**

Literature search was conducted using four databases, including gray literature, with additional manual search of the references of selected articles. Eligibility criteria consisted of randomized clinical trials, without restriction regarding the date or language of the publication. Two independent reviewers performed the article selection and data extraction. The random-effects meta-analysis used a non-standard technique to assess the mean difference in days to achieve full enteral nutrition, carried out by the Stata 15 statistic program.

**RESULTS:**

The systematic review comprised 10 studies, and five were selected for meta-analysis, with a population of 764 VLBW-PTNB and gestational age of birth between 25 and 32 weeks. The studies were conducted between 2011 and 2018 in North America, Asia and Africa, with only one conducted in South America. Altogether, they reported the number of days it took 708 VLBW-PTNB to achieve full enteral nutrition, with newborns treated with immunotherapy showing a shorter time in only three studies. Meta-analysis showed a mean difference of -4.26 days, (95% CI -7.44; -1.08d), with high heterogeneity (I^2^ = 83.1%).

**CONCLUSION:**

The use of oropharyngeal colostrum immunotherapy can reduce the time for VLBW-PTNB to achieve full nutrition when compared to those who used a placebo or received routine care.

## INTRODUCTION

Nutrition is essential for the newborn’s proper growth and development, especially for very low birth weight preterm newborns (VLBW-PTNB)^1^. The diet for this age group poses a nutritional emergency and great challenge since it must supply some nutrients equivalent to those they would possibly be receiving via the intrauterine route^2^.

Dietary nutrients are also essential for the maturation and development of gastrointestinal function and the installation of a healthy microbiota. Colostrum is rich in immunomodulatory bio-factors, stimulates the cells of the lymphoid tissues, and can favor the maturation of the immune system and the gastrointestinal tract^3^.

Oropharyngeal colostrum immunotherapy is a strategy than can provide antimicrobial and anti-inflammatory protective factors, a true immunological and trophic support^4^, condition needed to reduce the time between the use of parenteral (tube) feeding and full enteral nutrition (intake of 100 to 150 mL.kg^1^.day^1^)^5^. Such strategy would offer the VLBW-PTNB the nutrients required for their physical and neurological development^6^.

Although scientific evidence of the positive effect of colostrum on neonatal clinical outcomes^3,4,7,8^is available in the literature, no study has investigated colostrum immunotherapy associated with full nutrition as a primary outcome. Three randomized controlled clinical trials conducted with VLBW-PTNB have reported that they achieved full nutrition quicker in the colostrum treatment group compared to the control group^6,8,9^. However, other works did not mention the same association^10^.

Likewise, the meta-analyses that investigated the effect of oropharyngeal colostrum immunotherapy on morbimortality prevention^13^did not assess the time it took preterm newborns to achieve full enteral nutrition as a primary outcome of interest or did not research this outcome^16^.

Given the need for quality scientific evidence and the limited number of studies regarding the association in question, the current meta-analysis systematically investigated the effect of oropharyngeal colostrum immunotherapy in reducing the time VLBW-PTNBs take to achieve full enteral nutrition. This outcome can contribute to the newborns’ proper growth, development, shorter stay in intensive care, and to reduce neonatal morbimortality.

## METHODS

This meta-analysis used the PRISMA standard and included the following steps: registration and protocol; study eligibility criteria; database search; definition of search strategies; data selection, extraction and analysis; and qualitative assessment of the studies.

### Registration and Protocol

We searched for existing systematic reviews on the topic, with no record found. A review protocol was established before its completion and recorded in PROSPERO under number CRD42019126088. The protocol had no significant deviations during the research.

### Study Eligibility Criteria

Eligibility criteria consisted of randomized clinical trials evaluating the effect of oropharyngeal colostrum immunotherapy over the time it took VLBW-PTNBs (gestational age up to 37 weeks and weight below 1,500 g) to achieve full enteral nutrition. We applied no restriction concerning date or language of publication. Non-randomized clinical trials, cohort studies, and narrative and systematic literature reviews were excluded.

### Database Search

We conducted the literature search until July 31, 2020, in the following electronic databases: Medline/PubMed, Lilacs/Bireme/BVS, Cochrane Central, and Web of Science. Gray literature comprised: CAPES/MEC Portal thesis database, ProQuest Dissertations & Theses Databases, Clinical Trials study protocols, study protocols from the Brazilian Registry of Clinical Trials and contact with study authors with unpublished results. The references of the articles selected for the systematic review were also searched.

### Search Strategies

We used MeSH descriptors and their synonyms for searching Medline and Cochrane Central, and DeCS descriptors for the Lilacs/Bireme database. The terms in both languages (English and Spanish) were combined using *and* to ensure a good return of information in Lilacs and Bireme/BVS. To increase search sensitivity in the Medline database, our search strategy included the so-called “entry terms” or Boolean operators within the definition of the MeSH term. Table 1 presents the search strategies used for each database.

**Table 1:** Search strategies with keywords and Boolean operators used in different electronic databases.

Database	Strategy	n	Date
Cochrane	colostrum in Title Abstract Keyword AND Infant, Very Low Birth Weight in Title Abstract Keyword - (Word variations have been searched)	38	20Jul2020
Lilacs	(tw:(infant, very low birth weight)) AND (tw:(colostrum))	68	20Jul2020
Clinical trials	COLOSTRUM | Interventional Studies |	37	20Jul2020
Pubmed	(infant, low birth weight) AND colostrum	87	20Jul2020
Web of Science	(colostrum) AND TÓPICO: (Infant, very low birth weight)	66	20Jul2020
Google Acadêmico	Imunoterapia orofaríngea de colostro	36	20Jul2020
Portal CAPES/MEC	colostrum AND infant, low birth weight	24	20Jul2020
Proquest	colostrum AND Infant, very low birth weight	40	20Jul2020
Rebec	COLOSTRUM	2	20Jul2020

Source: Original of this manuscript.

### Study Selection

Two independent reviewers (MSXR and CCM) selected the studies by reading the titles and abstracts. Then, the same two researchers independently read the full text of the individually selected papers. Works that met the eligibility criteria were included in the systematic review. Any disagreement between the researchers on the inclusion or exclusion of a paper was settled by consensus (MSXR, CCM).

### Data Extraction

Two independent researchers (MSXR and CCM) extracted the data and later adjudicated them by consensus (Table 2). The data were entered in an Excel spreadsheet containing the following fields: type of publication, author, year of publication, title, objective, analysis method, results, conclusions, name and rating (Qualis) of the journal, research year, duration of follow-up, research funding, country of study, continent of study, inclusion and exclusion criteria, sample size, mean age of participants, mean age standard deviation, description of intervention and control, duration of oropharyngeal colostrum immunotherapy, time needed to achieve minimum enteral nutrition and full enteral nutrition, confounding variables and conflict of interest.

**Table 2:** General characteristics of the studies.

Authors/Year/ Location	Type of study / Funding source*	Sample size / n / group	Oropharyngeal colostrum immunotherapy protocol	Control group	Methodological quality of studies (Rob 2)	Time to achieve full enteral nutrition (days)
Lee et al.^10^ (2015) Seoul, Korea	Randomized controlled clinical trial * Seoul National University Medical School	48 Colostrum group (n = 24) Placebo group (n = 24) p = 0.86	0.2 mL of maternal colostrum was administered via oropharynx, 0.1 mL into the right and left oral mucosa every 3 hours after 48 to 96 hours of life, for 72 hours, regardless of the child’s enteral feeding.	0.2 mL of sterile water was administered, according to the oropharyngeal colostrum immunotherapy protocol.	Low risk	Colostrum group: 20 (13–27) Placebo group: 17 (14.3–25.8)
Rodriguez et al.^8^ (2011) Midwest, EUA	Randomized controlled clinical trial * Not informed	15 Colostrum group (n = 7) Placebo group (n = 6) p = 0.032	0.2 mL of maternal colostrum was administered via oropharynx, 0.1 mL into the right and left oral mucosa before 48h of life every 2h for 48h, enteral feeding was started after the protocol was completed.	0.2 mL of sterile water was administered, according to the oropharyngeal colostrum immunotherapy protocol.	Some concern	Colostrum group: 14.29 ± 5.74 Placebo group: 24.17 ± 8.66
Sohn et al.^7^ (2015) California, USA	Randomized controlled clinical trial * Not informed	12 Colostrum group (n = 6) Control group (n = 6) p = not statistically different	0.2 mL of maternal colostrum was administered into the oral cavity (0.1 mL on each side of the oral cavity) every 2 hours for 46 hours, regardless of whether the child was receiving trophic food.	Control group received routine care.	High risk	Colostrum group: 17(14–41) Control group: 13 (9–24)
Glass et al.^11^, (2017) Pennsylvania, USA	Randomized controlled clinical trial * Not informed	30 Colostrum group (n = 17) Sterile water group (n = 13) p = not statistically different	0.2 mL of maternal colostrum was administered to the oral mucosa with a swab every 3 hours for 2 to 7 days.	0.2 mL of sterile water was administered, according to the oropharyngeal colostrum immunotherapy protocol.	High risk	Colostrum group: 24.2 ± 7.9 Sterile water group: 24.9 ± 9.4
Romano-Keeler et al.^21^, (2016) Nashville, Tennessee, USA	Randomized controlled clinical trial * Thrasher Research Fund, USA	99 Colostrum group (n = 48) Non colostrum group (n = 51) p = 0.39	0.2 mL of maternal colostrum was administered, started in the first 48 hours of life, 0.1 mL on each side of the oral mucosa every 6 hours for 5 days.	Control group received routine care.	High risk	Colostrum group: 11 (8–15) Non colostrum group: 11(9–19)
Zhang et al.^6^, (2017) Shanghai, China	Randomized controlled clinical trial * Not informed	55 Colostrum group (n = 27) Control group (n = 28) p = 0.09	0.2 mL of maternal colostrum was administered, 0.1 mL on each side of the oral mucosa with a constant speed of at least 20 seconds over 4 hours, for seven days.	0.2 mL of saline solution was administered according to the oropharyngeal administration protocol.	Some concern	Colostrum group: 24.71 ± 11.23 Control group: 32.72 ± 20.11
Abd-Elgawad et al.^9^, (2019) Egypt	Randomized controlled clinical trial * Not informed	200 Colostrum group (n = 100) Gavage group (n = 100) p < 0.01	0.2 mL of maternal colostrum was administered to the oral mucosa, involving the oropharynx, tongue, and cheeks every 2 or 4 hours, during the pre-feeding period. When infants met the criteria for starting enteral feeding, 0.2 mL of the mother’s colostrum was administered to the oropharynx, tongue, and cheeks 5 minutes before gavage until Rn reached complete enteral feeding.	Nothing was administered during the pre-feeding period in the regular gavage group (control). The mother’s colostrum or breast milk was administered via tube when the premature infants adjusted to the criteria to start enteral feeding.	Some concern	Colostrum group: 11.10 ± 2.1 Gavage group: 15.57 ± 1.9
Sharma et al.^12^, (2020) India	Randomized controlled clinical trial * Not informed	117 Colostrum group (n = 59) Control group (n = 58) p = 0.61	Oropharyngeal administration of 0.2 mL of maternal colostrum to the oral mucosa, 0.1 mL directed to the oropharynx on both sides. Started after 24 hours of life every 2 hours for 72 hours, regardless of the infant’s enteric feeding status.	Control group newborns received routine care.	High	Colostrum group: 10.1 ± 5.7 Control group: 10.7 ± 4.3
Shiney Easo et al.^23^ (2018) Kuwait	Randomized controlled clinical trial * Not informed	43 Colostrum group (n = 21) Control group (n = 22) p = 0.888	0.1 mL of colostrum or fresh or chilled breast milk was administered slowly to the posterior end of the oral cavity, drop by drop over 30 s. Procedure was repeated on the opposite side. Therapy was started right after birth, every 4 hours, and continued until reaching complete enteral feeding.	Control group received 0.2 mL of sterile water applied in the same way and frequency as the intervention group. Started in the first 6 hours of life.	Some concern	Colostrum group: 16 (10–25.5) Control group: 16 (11–22)
Ferreira et al.^22^ (2019) Brazil	Randomized controlled clinical trial * The Minas Gerais Research Support Foundation Brasil	145 Feeding achieved 100 mL/kg/day Colostrum group (n = 47) Placebo group (n = 66) p = 0.45 Feeding achieved 150 mL/kg/day Colostrum group (n = 47) Placebo group (n = 66) p = 0.44	0.2 mL of maternal colostrum was administered in the first 48 or 72 hours of life every 2 hours for 48 hours, 0.1 mL to the right oral mucosa, and 0.1 mL to the left.	0.2 mL of sterile water was administered, according to the oropharyngeal colostrum immunotherapy protocol.	Some concern	Feeding achieved 100mL. kg^-1^.day^-1^ Colostrum group: 16 (13–22) Placebo group: 18 (15–20) Feeding achieved 150mL. kg^-1^.day^-1^ Colostrum group: 20 (18–26) Placebo group: 24 (18–25)

Note: The time to reach full enteral nutrition (days) was presented as median (interquartile range) in studies 10, 7, 21, 23 and 22 and on mean ± standard deviation in studies 8, 11, 6, 9 and 12. Source: Original of this manuscript.

### Study Quality Assessment

To assess the quality of the selected studies, we used the Cochrane risk-of-bias tool for randomized trials (RoB 2)^18^.

### Data Analysis

After the pre-selection (by two reviewers) of the studies as per the inclusion criteria using the StArt (State of the Art through Systematic Review) tool, with a third reviewer consulted to achieve consensus, the researchers prepared a summary of the qualitative data of the included studies comprising the characteristics of the investigations. Statistical heterogeneity was assessed using I^2^; depending on the magnitude of this indicator, we performed a meta-analysis^19^ and assessed the magnitude of the inconsistency. The random-effects meta-analysis used nonstandard technique to assess the mean difference in days to achieve full enteral nutrition^20^. Weighted mean difference was used to combine the effect size estimates of the study. The estimate of the combined effect in this method represents a weighted average of all studies that included group comparisons.

For group comparisons in the analysis, each individual result is assigned a weight inversely proportional to the variance; thus larger trials are assigned more weight, and smaller trials, less weight. The analysis was conducted using Stata 15 statistic program.

## RESULTS

### Selected Studies

**Figure 1:**
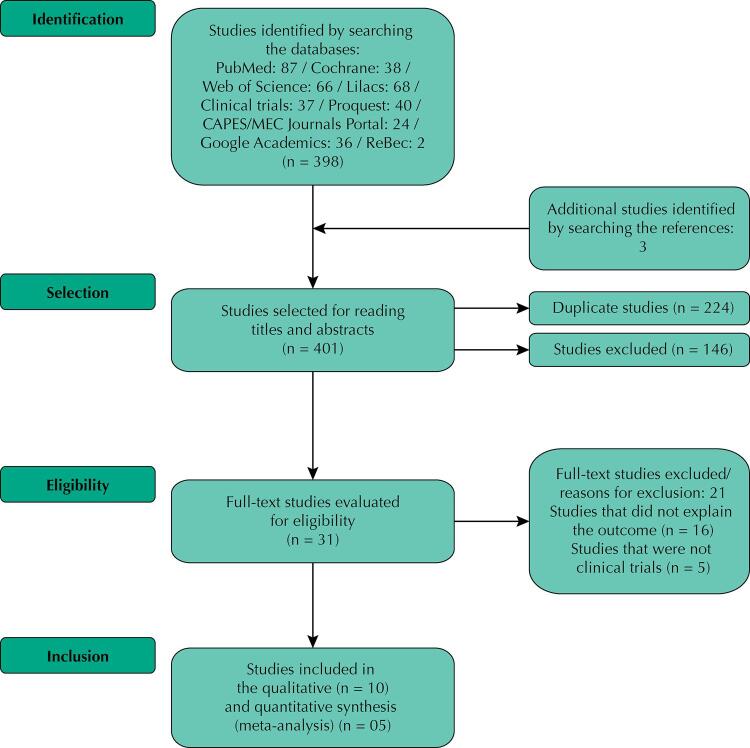
Flowchart of the systematic literature review.

The literature search identified 398 articles, with three manually added after examining the references of the selected papers, totaling 401 studies for reading titles and abstracts. This first screening removed 224 duplicates and excluded 146 studies that did not meet the topic of interest, leaving 31 papers to be read in full. After this second reading, we excluded 21 studies: 16 for not describing the outcome of interest, and five for not being a randomized clinical trial. Only 10 studies met the eligibility criteria for this systematic review (Figure 1). The publication period for the included investigations was until July 2020.

### General Characteristics and Quality of the Studies

All the studies included in this review were randomized clinical trials, comprising a study population of 764 VLBW-PTNB, with gestational age between 25 and 32 weeks, weighing below 1,500 g. The surveys were conducted from 2011 to 2018, with most papers conducted in North American, Asian, and African countries, and only one South American country.

All the included studies aimed to evaluate the efficacy of oropharyngeal colostrum immunotherapy on outcomes related to some health condition of newborns by comparing the administration of oropharyngeal colostrum versus the use of placebo (sterile water or saline solution) or routine care. Nine studies were published^6^ and one was described in an unpublished report provided by its author^23^.

The therapeutic clinical protocol in the analyzed studies consisted in administering 0.2 mL of colostrum in the oropharynx of newborns, 0.1 mL in the right oral mucosa and 0.1 mL in the left mucosa with sterile tuberculin syringes, except for two studies: one, which used the gentle cleansing technique with a swab inside the mouth, cleaning cheeks, tongue, palate and lips; and another, which administered colostrum by gavage.

One of the studies used milk from the milk bank in the treatment group, when raw colostrum was unavailable^22^. The time of colostrum administration ranged from 48 hours to seven days, with two clinical protocols applying the therapy until the newborns reached full enteral nutrition.

Regarding the control group, six studies used the same administration technique as that of the intervention group, differing only in the use of a placebo (sterile water or saline solution)^6^. In the other four studies, the control group remained under the routine care at the hospital unit^7,9,12,21^.

As for the methodological quality of the studies, assessed by the Cochrane risk-of-bias tool for randomized trials (RoB 2)^18^, we identified variable quality among all studies: one showed low risk of bias^10^, five were classified with some concern^6,8,9,22,23^, and the remaining four as a high risk of bias^9,11,12,21^.

All studies reported random allocation of subjects, but two of them did not specify the method used to generate the random sequence^7,8^. Concerning the concealment method, four studies reported blinding the participants^6,10,22,23^ by opaque envelopes and sealed syringes, three studies did not report blinding^7,8,11^, and three reported not being blind^9,12,21^.

Seven studies obtained the data assessed for outcomes from all participants, while three studies assessed only some of the participants^6,8,23^. The analysis considered all the methods used for measuring outcomes as appropriate. Altogether, the studies reported days for complete enteral feeding in 708 VLBW-PTNB. Three of the studies included fewer participants in assessing the time to achieve full enteral nutrition^6,8,23^.

**Figure 2:**
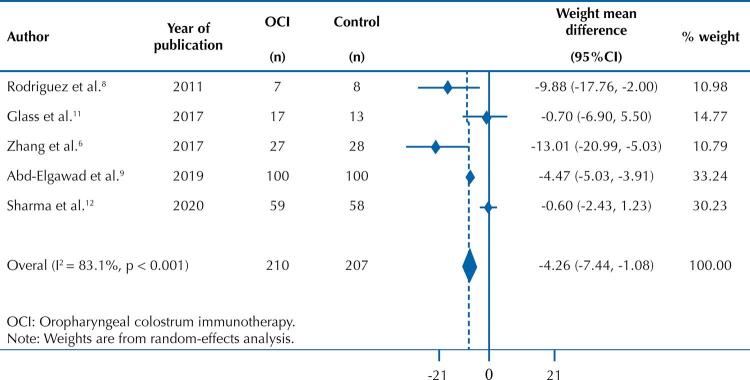
A random-effects meta-analysis of oropharyngeal colostrum immunotherapy over time to achieve full enteral nutrition in very low birth weight preterm infants.

Only three studies reported the faster establishment of full enteral nutrition in the VLBW-PTNB who received oropharyngeal colostrum immunotherapy^6,8,9^. Five studies showed means and standard deviation in days for newborns to achieve full nutrition, and the remaining five showed the results by median and interquartile range. Thus, we selected only five studies for the meta-analysis^6,8,9,11,12^ (Figure 2), as they had the information required to generate a summary measure of mean difference in days to achieve full enteral nutrition.

The five studies included in this meta-analysis reported the oropharyngeal colostrum immunotherapy effect over time to achieve full enteral nutrition in 417 VLBW-PTNB. All five papers estimated the outcome analyzed by mean and standard deviation measures. Three of these studies showed that the colostrum treated group reached full enteral nutrition more quickly^6,8,9^, whereas the other two found no statistically significant difference when comparing the treated group with the control group^11,12^.

Our meta-analysis of the five studies found a mean difference of -4.26 days, (95% CI: -7.44; -1.08d), showing that the use of oropharyngeal colostrum immunotherapy can reduce the time to achieve full nutrition in VLBW-PTNB when compared to those treated with placebo or that received routine care. However, we found a high heterogeneity between these studies (I^2^= 83.1%), and the low number of papers identified for this meta-analysis hindered its adjustment.

## DISCUSSION

The present meta-analysis found that VLBW-PTNB on oropharyngeal colostrum immunotherapy required less time to achieve full enteral nutrition, a result that reinforces the recommendation for using colostrum as an immunological therapy.

Such an intervention is expected to shorten the premature infants’ time to achieve full enteral nutrition, since colostrum provides functional nutrients and bioactive components that favor a microenvironment for the defense and maturation of the intestinal mucosa^24,25^ as well as a colonization of the gastrointestinal tract by enteric bacteria with antibacterial functions, immunomodulation, and production of nutritional metabolites, which characterize a healthy microbiome^26^.

Early feeding practices of preterm infants are a potentially modifiable risk factor. Previously, observational studies suggested that conservative diets with slow volume advancement would reduce the risk of necrotizing enterocolitis. However, recent research has shown that slow feeding progress can delay the establishment of full enteral feeding, and may be associated with metabolic and infectious morbidities secondary to prolonged exposure to parenteral nutrition^27^.

Despite its biological plausibility, the quality of the evidence identified in this meta-analysis was low due to the high heterogeneity of the included studies, and the large confidence interval (-7.44 to -1.08d). Some methodological characteristics of the individual studies may have contributed to a possible change in the global measure and high heterogeneity, such as small sample size^6,8,10,11,21,23^, variable clinical protocols, and time defined to reach full enteral nutrition (100 to 150 mL.kg^-1^.day^-1^). When evaluating the studied variables, we identified the inclusion of different birth weight values^6,9,20^ and Apgar scores^8,11,12^.

In all studies analyzed, enteral feeding started according to the hospital’s protocol or according to individualized treatment for each newborn, with no standardization. Some studies recorded feeding initiation in the first 24 hours after live birth^9,23^ or more than 24 hours^7^. These feeding protocols used breast milk, when produced in sufficient quantity^6^, donor milk^21^, or formula^9,10,21^. Regarding the number of doses of colostrum administered during treatment, one study^8^ registered the administration of 75 to 85% of the doses planned for treatment, another^6^ noted that 42 doses were administered during the 7-day therapy, and a third research^7^ reported the administration of 24 doses per newborn.

Regarding biases, we observed a predominant selection bias^6,8,9,11,12,20,21,23^, specifically for allocation concealment. The analysis found some weaknesses in the text description regarding the information necessary to assess the other bias domains^6,8,9,12^, which can cause essential distortions in the global measure. For example, we identified no use of adjustment for confounder covariables in the primary studies. The present meta-analysis could not assess the publication bias due to the insufficient number of selected studies. Even considering the methodological weaknesses of the included studies, they were kept in the analysis given the low number of papers on the topic.

A meta-analysis on the use of this procedure in preventing morbimortality in premature infants, found an association between the use of oropharyngeal colostrum immunotherapy and the rapid achievement of full enteral nutrition. It found very low quality of evidence due to imprecision, high risk of bias, and moderate heterogeneity of the included studies^13^.Two other meta-analysis that studied the effect of this therapy on necrotizing enterocolitis^15^ and prematurity^14^, found no significant results for this association.

Importantly, these previous meta-analyses are grounded on some methodological characteristics recommended by other researchers^28,29^, such as the estimation of the mean and standard deviation by medians and interquartile ranges among the reviewed studies^13^.

These methods are questionable, since by assuming that the mean can be estimated from the median, without incorporating the influence of the sample size, this ignores data distribution regarding the principle of normality. It is not reasonable, for example by the method used by Hozo et al. 2005^29^, to consider the equivalence between mean and median values for data that present asymmetry in their distribution^30^. Therefore, we understand that the previous meta-analyses on the topic in question present methodological weaknesses^31^.

The present meta-analysis differs from those mentioned as it included only randomized clinical trials with results related to means and standard deviation, without estimating the measures presented in the individual studies. Ours is the second study to have judiciously evaluated the methodological quality of studies using the Rob2 tool^18^, and the first to have investigated exclusively the time to achieve full enteral nutrition in very low birth weight premature newborns, giving visibility to a clinical outcome relevant to their growth and development.

## CONCLUSIONS

Our findings show that the use of oropharyngeal colostrum immunotherapy can reduce the time to achieve full enteral nutrition in VLBW-PTNB when compared to those treated with placebo or routine care. Achieving full enteral nutrition more quickly and without undesirable effects is essential to provide good growth and development for premature infants.

For a better quality of scientific evidence, further studies with standardized colostrum oropharyngeal therapy protocols that explore the time of onset, the dose to be administered, and the frequency and duration of treatment are needed.

Finally, it is important to highlight the importance of colostrum immunotherapy and early and adequate nutrition for these newborns’ health in preventing possible unfavorable clinical outcomes, achieving survival with quality of life, and avoiding sequelae in the medium and long term.
